# Flexible Krylov Methods for Edge Enhancement in Imaging

**DOI:** 10.3390/jimaging7100216

**Published:** 2021-10-18

**Authors:** Silvia Gazzola, Sebastian James Scott, Alastair Spence

**Affiliations:** Department of Mathematical Sciences, University of Bath, Bath BA2 7AY, UK; ss2767@bath.ac.uk (S.J.S.); masas@bath.ac.uk (A.S.)

**Keywords:** flexible Golub–Kahan decomposition, iteratively reweighted least squares, edge enhancement, image deblurring, image inpainting, computed tomography

## Abstract

Many successful variational regularization methods employed to solve linear inverse problems in imaging applications (such as image deblurring, image inpainting, and computed tomography) aim at enhancing edges in the solution, and often involve non-smooth regularization terms (e.g., total variation). Such regularization methods can be treated as iteratively reweighted least squares problems (IRLS), which are usually solved by the repeated application of a Krylov projection method. This approach gives rise to an inner–outer iterative scheme where the outer iterations update the weights and the inner iterations solve a least squares problem with fixed weights. Recently, flexible or generalized Krylov solvers, which avoid inner–outer iterations by incorporating iteration-dependent weights within a single approximation subspace for the solution, have been devised to efficiently handle IRLS problems. Indeed, substantial computational savings are generally possible by avoiding the repeated application of a traditional Krylov solver. This paper aims to extend the available flexible Krylov algorithms in order to handle a variety of edge-enhancing regularization terms, with computationally convenient adaptive regularization parameter choice. In order to tackle both square and rectangular linear systems, flexible Krylov methods based on the so-called flexible Golub–Kahan decomposition are considered. Some theoretical results are presented (including a convergence proof) and numerical comparisons with other edge-enhancing solvers show that the new methods compute solutions of similar or better quality, with increased speedup.

## 1. Introduction

In this paper, we consider the solution of large-scale linear systems of the form

(1) $$Ax_{\mathrm{true}}+e=b_{\mathrm{true}}+e=b\;.$$

We are interested in problems (1) associated with the discretization of linear inverse problems, where $b\in R^{m}$ represents the measured data, $A\in R^{m\times n}$ represents the forward mapping, $x_{\mathrm{true}}\in R^{n}$ is the desired solution, and $e\in R^{m}$ is unknown Gaussian white noise. In this setting, *A* is typically ill-conditioned with ill-determined rank (i.e., the singular values of *A* decay and cluster at zero without an evident gap between two consecutive ones). Systems such as (1) are central in many imaging problems, including image deblurring, image inpainting, and computed tomography, where the matrix *A* represents convolution, a combination of undersampling and convolution, and discrete Radon transform, respectively; see [1,2,3]. In this framework, $x_{\mathrm{true}}\in R^{n}$ is a vectorialization of a 2D image $X_{\mathrm{true}}\in R^{N\times N}$, with $N=\sqrt{n}$, obtained, for instance, by stacking the columns of $X_{\mathrm{true}}$; we compactly denote this operation by $x_{\mathrm{true}}=\mathrm{vec}\left(X_{\mathrm{true}}\right)$ and its inverse by $X_{\mathrm{true}}={\mathrm{vec}}^{-1}\left(x_{\mathrm{true}}\right)$.

Due to the ill-conditioning of *A* and the presence of noise *e* in (1), some regularization must be applied in order to compute a meaningful approximation of $x_{\mathrm{true}}$. Although many efficient iterative methods are routinely used to regularize (1) by early termination of the iterations (see, e.g., [4,5,6,7] and the references therein), in this paper, we consider a variational regularization method to compute

(2) $$x_{\mathrm{reg}}=\arg\min_{x\in R^{n}}{∥Ax-b∥}_{2}^{2}+\hat{\lambda }\Omega \left(x\right)\;,$$

where Ω(x) is a problem-specific regularizer, chosen to enforce a priori information about $x{}_{\mathrm{true}}$ onto the regularized solution $x{}_{\mathrm{reg}}$, and $\hat{\lambda }>0$ is regularization parameter that specifies the amount of regularization to be imposed. Common and somewhat basic choices for the penalty term include $\Omega \left(x\right)=*x_{2}^{2}$ and $\Omega \left(x\right)=*{Lx}_{2}^{2}$ with $L\in R^{p\times n}$, corresponding to standard and generalized Tikhonov regularization, respectively. Although such choices reduce (2) to a quadratic problem, two drawbacks arise when 2-norm regularization is applied to solve inverse problems in imaging, where *A* is typically unstructured and large-scale: firstly, an iterative solver must be employed to compute $x_{\mathrm{reg}}$ (see [1,4,5,8] and the references therein); secondly, $x_{\mathrm{reg}}$ may be inherently over-smoothed and therefore unsuitable when edge information should be accurately recovered (see [2]). To overcome the second drawback, one should resort to functionals Ω(x) involving some *q*-(quasi)norm, 0<q≤1, and solve (2) using appropriate optimization methods: there is a rich body of literature about this, and we point to [9] for a recent survey.

In this paper, we consider the edge information that is revealed by computing the gradient of an image, and, in this setting, one of the most popular edge-enhancing regularizers is total variation (TV) [10]. In its original form and in a discrete setting, the TV of a vector *x* measures the magnitude of the discrete gradient of *x* in the $\ell_{1}$ norm; recall that, in this paper, x=vec(X). Therefore, considering TV as a regularizer has the effect of allowing a few (possibly steep) changes in the gradient of $x_{\mathrm{reg}}$ or, equivalently, solutions with a sparse gradient. TV-like functionals, which may penalize the gradient in the horizontal and vertical directions separately, or use a variety of norms for evaluating the gradient to enforce even more sparsity, have also been considered. Among the most popular solvers for TV regularization, we list proximal gradient methods, hybrid primal–dual methods, split Bregman methods, and iteratively reweighted norm methods; we refer to [11,12,13,14,15,16].

In this paper, we focus on the class of iteratively reweighted least squares (IRLS) solvers, also called iteratively reweighted norm (IRN) solvers, associated with TV-like and edge-enhancing functionals. IRLS methods solve (approximately) a sequence of reweighted, penalized, least squares (LS) problems that are increasingly improved approximations of (2). Consider the reformulation of (2) as a nonlinear optimization problem of the form

(3) $$x_{\mathrm{reg}}=\arg\min_{x\in R^{n}}{∥Ax-b∥}_{2}^{2}+\lambda {∥W\left(Lx\right)Lx∥}_{2}^{2}\;,\;\lambda =\frac{\hat{\lambda }}{2}\;,$$

where $L\in R^{p\times n}$ and $W\left(Lx\right)\in R^{p\times p}$ is a diagonal weighting matrix whose entries depend on Lx. Formulating (2) (or a smooth approximation thereof) as (3) is quite straightforward when, e.g., $\Omega \left(x\right)={∥Lx∥}_{q}^{q}$; see [17]. Starting from an initial approximation $x_{0,★}$ of $x_{\mathrm{true}}$, the IRLS method solves (approximately) a sequence of quadratic problems of the form

(4) $$x_{k,★}=\arg\min_{x\in R^{n}}{∥Ax-b∥}_{2}^{2}+\lambda {∥W_{k}Lx∥}_{2}^{2}\;,\;k=1,2,\ldots ,\;\mathrm{where}\;W_{k}=W\left(Lx_{k-1,★}\right),$$

and convergence of $x_{k,★}$ to $x_{\mathrm{reg}}$ is guaranteed under mild assumptions; see, e.g., [18,19] and the references therein. All but one of the methods considered in this paper can be reformulated as (3). The specific expressions of the matrices W(Lx), $W_{k}$, and *L* appearing in (3) and (4) depend on the choice of TV-like regularizer, and will be detailed in Section 2. We also mention that some IRLS schemes (4), including one considered later in this paper, are not necessarily associated with a variational formulation of the kind in (2) and (3); see [20,21] for more details. If the null spaces of *A* and $W_{k}L$ intersect trivially, problem (4) has the unique solution

$$x_{k,★}={\left(A^{T}A+\lambda L^{T}W_{k}^{T}W_{k}L\right)}^{-1}A^{T}b\;.$$

However, as hinted at the beginning of this section, for large-scale unstructured problems (1), it is too demanding to compute $x_{k,★}$ directly and, therefore, an iterative method (usually a Krylov projection method, which relies on the computation of matrix–vector products with *A*, $W_{k}L$, and often their transposes) should be employed to approximate $x_{k,★}$. As a consequence, classical IRLS methods unavoidably rely on inner–outer iterative schemes, where the outer iteration updates the weights $W_{k}$, while the inner iteration solves each (iteratively reweighted) least squares problem. To the best of our knowledge, an IRN approach for TV was first proposed in [22], where an expression for the edge-enhancing weights was first derived; more specifically, the authors of [22] consider a fixed regularization parameter and solve each least squares problem in the sequence by the conjugate gradient method. In a similar setting (stemming from the lagged diffusivity fixed point iteration [11]), the authors of [12] propose to use the so-called ‘modified’ LSQR method to solve efficiently each least squares problem in the sequence after performing a change of variable that involves the ‘inversion’ of the matrix $L^{T}W_{k}^{T}W_{k}L$, with the added benefit of adaptive regularization parameter choice.

Recently, novel solvers for IRLS have been proposed, which approximate the solution of (2) by avoiding nested iteration cycles. This is possible by updating the weights $W_{k}$ as soon as a new approximate solution becomes available—namely, immediately after a new iteration of a solver for the iteratively reweighted LS problem (4) is computed—and by employing modified Krylov projection methods that can handle changes in the LS problem (specifically, changes in the weights). Such solvers are based either on generalized Krylov subspace (GKS) methods (see [19,23]), or on flexible Krylov subspace (FKS) methods (see [17,24,25]). Rather than computing the solution $x_{k,★}$ as in (4), the *k*th iteration of a GKS method computes an approximation $x^{\left(k\right)}$ to $x_{\mathrm{reg}}$ by projecting problem (4) onto a so-called ‘generalized Krylov subspace’ of dimension *k*, which is then extended in the direction of the residual $\left(A^{T}A+\lambda L^{T}W_{k}^{T}W_{k}L\right)x^{\left(k\right)}-A^{T}b$. Such methods can be efficiently applied to a variety of regularization terms of the form $\Omega \left(x\right)={∥Lx∥}_{q}^{q}$, provided that matrix–vector products with *L* are cheap to compute. To project problem (4) onto the current approximation subspace, it may be necessary to compute economy-size QR decompositions of m×k and p×k matrices, but this is not demanding when k≪min{n,p}. Flexible Krylov methods can be applied only after problem (4) has been transformed into standard form [26]—namely, after a change of variables has been applied and (4) has been reformulated as an equivalent Tikhonov problem with a regularization term of the form $\Omega \left(x\right)={∥x∥}_{2}^{2}$ and with *A* replaced by an operator that includes the action of the (‘inverted’) matrix $W_{k}L$. Sometimes, the ‘inversion’ of the matrix $W_{k}L$ is referred to as ‘priorconditioning’ because of its connection with a Bayesian approach to inverse problems [27]. More details about this process are provided in Section 3. In particular, the *k*th iteration of a flexible Krylov method generates an approximation subspace of dimension *k* that incorporates the action of the ‘inverted’ weights $W_{i}$, i=1,…,k, and its efficiency depends on the considered regularizer and the cost associated with the ‘inversion’ of $W_{k}L$. For a variety of regularization terms (see, e.g., [24,25,28]), this can be done with negligible computational overhead. Convergence of both GKS and FKS methods can be proven by resorting to the framework of majorization–minimization (MM) methods [29]. Both GKS and FKS methods allow adaptive (i.e., iteration-dependent) regularization parameter choice, which is crucial in the common scenario where a good value of λ is not known a priori. Indeed, allowing some heuristic arguments, a suitable value of the regularization parameter can be efficiently chosen at the *k*th iteration by manipulating a reduced-size projected problem, rather than having to solve the original problem (2) multiple times, one for each value of λ (preset by the user or dictated by the application of some parameter choice rule); more details about these approaches are provided in Section 4. A review of GKS and FKS methods for regularization is available in [18].

This paper aims at introducing new solvers for (2) based on the flexible Golub–Kahan (FGK) decomposition [30], introducing significant elements of novelty with respect to available solvers based on either FKS or GKS methods. Firstly, while ways of handling ‘sparsity under transform’ regularizers within a FKS framework were already presented in [24], these require an orthogonal ‘sparsity transformation’ (e.g., some choices of wavelets). The edge-enhancing regularizers considered in this paper are more general and more challenging to apply, as often the ‘sparsity transformation’ associated with the gradient of an image is rank-deficient and suitable strategies have to be devised to perform efficient computations, leading to a unified treatment of both the isotropic and anisotropic TV regularization terms, as well as other heuristic edge-enhancing regularizers. Similarly to the methods described in [24], convenient strategies to set the regularization parameter can be applied, resulting in inherently parameter-free solvers. We refer to Section 4 for more details. Secondly, although the idea of incorporating an edge-enhancing regularizer within a flexible Krylov method based on the flexible Arnoldi algorithm (i.e., FGMRES) was already proposed in [30], this is limited to the case of isotropic TV and a square matrix *A*; moreover, it is well-known that iterative solvers based on the Arnoldi algorithm are not general-purpose regularization methods and are only successful for matrices *A* close to normal or when the generated approximation subspace is favorable for a particular solution; we refer to [31] for more details about GMRES, which can be extended to FGMRES. We also note that, when adopting the method in [30], the regularization parameter should be set to 0 in the projected problem—this is not the case anymore when the new FGK-based solvers are considered. Lastly, while TV regularizers can be naturally handled by GKS-based methods [19,23], the approximation subspace for the solution generated by the new FGK-based solvers is potentially more efficient than the approximation subspace generated by GKS-based solvers, meaning that a high-quality solution can be recovered in smaller approximation subspaces; this is clearly visible in the numerical comparisons presented in Section 5.

The new FGK-based solvers are analyzed theoretically. Namely, a convergence proof is provided for the isotropic and anisotropic TV cases, and insight into the efficient approximate ‘inversion’ of all the considered regularizers is provided. Finally, extensive numerical experimentation (some of which is reported in this paper) shows that the new solvers are always able to compute regularized solutions of comparable or better quality, often with a great speedup, with respect to other edge-enhancing methods, such as the IRN and GKS strategies, and the proximal gradient solver. We refer to Section 5 for detailed comparisons.

This paper is organized as follows. Section 2 presents the regularization terms considered in this paper and the expressions for the edge-enhancing weighting matrices. Section 3 contains some background material, including a summary of the procedure for transforming problem (4) into standard form, and some details about FKS methods based on the flexible Golub–Kahan decomposition (FGK). Section 4 introduces the new edge-enhancing solvers based on FGK, and describes their properties and implementation details. Section 5 contains numerical experiments performed on three different imaging problems (involving deblurring, inpainting, and tomography). Section 6 presents some concluding remarks and possible future research directions.

## 2. Edge-Preserving Regularization via IRLS

As discussed in Section 1, to obtain a suitable reconstruction of $x_{\mathrm{true}}$, we require a problem-specific regularization term Ω(x) in (2) that enforces prior information on $x_{\mathrm{reg}}$. When $x_{\mathrm{true}}$ represents an image and when its edges should be preserved in $x_{\mathrm{reg}}$, a common choice for Ω(x) is the *q*-norm of (a function of) the gradient of *x* evaluated in some some *q*-(quasi)norm, 0<q≤1. We therefore open this section by defining a discrete gradient operator $D_{2d}$ for 2D images $X\in R^{N\times N}$, building upon the corresponding 1D operator. Let

$$D_{1d}=\left[\begin{array}{ccccccc} 1 & & -1 & & & & \\ & & \ddots & & \ddots & & \\ & & & & 1 & & -1 \end{array}\right]\in R^{(N-1)\times N}$$

be a scaled finite difference approximation of the first derivative operator. Then, the 2D arrays

$$D_{1d}X\in R^{(N-1)\times N}\;and\;{\left(D_{1d}X^{T}\right)}^{T}\in R^{N\times (N-1)}$$

contain the scaled first derivatives of *X* in the vertical and horizontal directions, respectively; see, e.g., [1]. These can be reshaped as 1D arrays using the vec(·) operation, and corresponding expressions for the discrete first derivative operators in the vertical and horizontal directions are obtained by exploiting well-known properties of the Kronecker product ⊗; see, e.g., [2]. Namely,

$$\begin{array}{ccccc} \mathrm{vec}(D_{1d}X) & = & \left(I⊗D_{1d}\right)\mathrm{vec}\left(X\right) & = & (I⊗D_{1d})x,\\ \mathrm{vec}(XD_{1d}^{T}) & = & \left(D_{1d}⊗I\right)\mathrm{vec}\left(X\right) & = & (D_{1d}⊗I)x\;, \end{array}$$

so that

$$D_{2d}=\left[\begin{array}{c} D^{v}\\ D^{h} \end{array}\right]=\left[\begin{array}{ccc} I\;\;\;\;\;\; & ⊗ & \;\;\;\;\;\;D_{1d}\\ D_{1d}\;\;\;\;\;\; & ⊗ & \;\;\;\;\;\;I \end{array}\right]\in R^{2\tilde{n}\times n}$$

is the scaled discrete 2D gradient operator, where $\tilde{n}:=N\left(N-1\right)$; the superscripts *v* and *h* stand for ‘vertical’ and ‘horizontal’ directions, respectively.

Before going into the specifics of the weights associated with the IRLS methods considered in this paper, we highlight that the following equalities and approximations will be extensively used. Let 0<q≤1, and let us consider the function $f_{q,\tau }$ defined for any vector $v\in R^{\ell }$, ℓ≥1, and a fixed τ (independent of *q*), as

(5) $$f_{q,\tau }\left(v\right)={\left({∥v∥}_{2}^{2}+\tau^{2}\right)}^{\frac{\left(q-2\right)}{4}}\;.$$

Given a vector $u\in R^{n}$, whose *j*th entry is denoted by ${\left[u\right]}_{j}$, we write

(6) $${∥u∥}_{q}^{q}=\sum_{j}{\left|{\left[u\right]}_{j}\right|}^{q}≃\sum_{j}\underset{=:{\left[w\left(u\right)\right]}_{j}^{2}}{\underset{︸}{f_{q,\tau }^{2}\left({\left[u\right]}_{j}\right)}}{\left({\left[u\right]}_{j}\right)}^{2}=u^{T}{\left(W(u)\right)}^{2}u=∥{W(u)u}_{2}^{2}∥\;.$$

Note that, in the expression above, $f_{q,\tau }\left({\left[u\right]}_{j}\right)={\left({\left[u\right]}_{j}^{2}+\tau^{2}\right)}^{q-2\;/\;4}$ avoids potential division by 0 and introduces some smoothness in the *q*-norm. The matrix $W\left(u\right)\in R^{n\times n}$ appearing in (6) is diagonal, and its (j,j)th entry is

$${\left[W\left(u\right)\right]}_{j,j}:={\left[w\left(u\right)\right]}_{j}:=f_{q,\tau }\left({\left[u\right]}_{j}\right)\;;$$

The dependency on the vector *u* and its *k*th entry is highlighted in the notations. In the following, we focus on the case q=1 and we describe the different edge-enhancing regularizers considered in this paper.

### 2.1. Isotropic Total Variation

As hinted in Section 1, isotropic total variation (TV) is a popular choice of regularization that penalizes the magnitude of the gradient in the $\ell_{1}$-norm. We adopt the following definition of discrete isotropic total variation:

(7) $$\mathrm{TV}\left(x\right):={∥{\left({\left(D^{v}x\right)}^{2}+{\left(D^{h}x\right)}^{2}\right)}^{1/2}∥}_{1}≃{∥W_{\mathrm{TV}}\left(D_{2d}x\right)D_{2d}x∥}_{2}^{2},$$

where the squaring and square root operations are applied entry-wise. Note that TV(x) as defined above is not invariant with respect to horizontal and vertical flips, nor for rotations of $\pm 90^{\circ }$, of the 2D image $X={\mathrm{vec}}^{-1}\left(x\right)$; see [32]. The weighting matrix for the rightmost smooth approximation in (7) reads

(8) $$\begin{array}{cc} \; & W_{\mathrm{TV}}\left(D_{2d}x\right)=\left[\begin{array}{cc} \left({\tilde{W}}_{\mathrm{TV}}\left(D_{2d}x\right)\right) & 0\\ 0 & \left({\tilde{W}}_{\mathrm{TV}}\left(D_{2d}x\right)\right) \end{array}\right]\in R^{2\tilde{n}\times 2\tilde{n}},\\ \; & \mathrm{where}\;{\tilde{W}}_{\mathrm{TV}}\left(D_{2d}x\right)=\mathrm{diag}\left(f_{1,\tau }\left([D^{v}x;D^{h}x]\right)\right)\in R^{\tilde{n}\times \tilde{n}}\;. \end{array}$$

Such weights are employed within an IRLS scheme as explained in (3) and (4), with $L=D_{2d}$.

### 2.2. Anisotropic Total Variation

Discrete anisotropic total variation (aTV) is defined as

(9) $$\mathrm{aTV}\left(x\right):={∥\left[\begin{array}{c} D^{v}\\ D^{h} \end{array}\right]x∥}_{1}≃∥{W_{\mathrm{aTV}}\left(D_{2d}x\right)D_{2d}x∥}_{2}^{2}\;,$$

where the weighting matrix for the rightmost smooth approximation is defined as

(10) $$\begin{array}{cc} \; & W_{\mathrm{aTV}}\left(D_{2d}x\right)=\left[\begin{array}{cc} \mathrm{diag}(w^{v}\left(D_{2d}x\right)) & 0\\ 0 & \mathrm{diag}(w^{h}\left(D_{2d}x\right)) \end{array}\right]\in R^{\tilde{n}\times \tilde{n}},\\ \; & \mathrm{where}\;\begin{array}{ccc} {\left[w^{v}\left(D_{2d}x\right)\right]}_{j} & := & f_{1,\tau }\left({\left[D^{v}x\right]}_{j}\right)\\ {\left[w^{h}\left(D_{2d}x\right)\right]}_{j} & := & f_{1,\tau }\left({\left[D^{h}x\right]}_{j}\right) \end{array},\;j=1,\ldots ,\tilde{n}. \end{array}$$

Such weights are employed within an IRLS scheme as explained in (3) and (4), with $L=D_{2d}$.

### 2.3. Edge-Enhancing Weights

A further choice of weighting matrix is related to the one originally introduced in [20], which is not associated with a variational formulation of the kind (2) nor to an analytical expression of the weights as in (7) or (9). Rather, such a weighting matrix relies on the cumulative effect of iteration-specific weights, whereby information from all previous iterates is retained. Specifically, assume that an estimate $x_{k-1,★}$ to $x_{\mathrm{true}}$ is obtained by (approximately) solving the (k−1)th instance of a IRLS problem that reads similarly to (4), with $W_{k}=W_{k}^{\mathrm{diag}}$ and $L=D_{2d}$. Here,

(11) $$\begin{array}{ccc} W_{k}^{\mathrm{diag}} & = & \mathrm{diag}\left(1-{\left(\frac{|W_{k-1}^{\mathrm{diag}}D_{2d}x_{k-1,★}|}{\;\;∥{W_{k-1}^{\mathrm{diag}}D_{2d}x_{k-1,★}∥}_{\infty }}\right)}^{a}+\tau \;\right)W_{k-1}^{\mathrm{diag}}\\ \; & =: & \mathrm{diag}(w_{k}^{\mathrm{diag}}) \end{array}\;,\;a>0,\;\tau >0\;\mathrm{fixed}.$$

In the above expression, 1 is a vector of all ones, and both the absolute value |·| and exponentiation are performed component-wise. The first weighting matrix $W_{0}^{\mathrm{diag}}$ may be either defined with respect to a sufficient initial guess $x_{0,★}$ (where at least one edge is visible), or simply taken to be the identity. The first diagonal term in $W_{k}^{\mathrm{diag}}$ updates the weights in such a way that the components of $W_{k-1}^{\mathrm{diag}}D_{2d}x_{k-1,★}\;/\;{∥W_{k-1}^{\mathrm{diag}}D_{2d}x_{k-1,★}∥}_{\infty }$ close to a dominant edge exclusively visible in the (k−1)th approximate solution are not penalized, while the oscillating components (i.e., those components that currently display spurious oscillations around a constant value) are penalized. The second diagonal term in $W_{k}^{\mathrm{diag}}$ encodes the edge information recovered at the previous iterations, so that also the dominant edges in the previous approximate solutions are not penalized, either. The parameter a>0 affects the amount of smoothness in the reconstructions; namely, choosing a≫1 results in more penalization of the supposedly smooth regions. The parameter τ>0 prevents singularities in the matrix $W_{k}^{\mathrm{diag}}$ (i.e., it has the same purpose as the parameter τ appearing in (5)).

## 3. Background Material: Standard Form Transformation and (Flexible) Krylov Solvers

As discussed in Section 1, when dealing with large-scale unstructured problems (1), each least squares problem of the form (4) arising within an IRLS method needs to be solved by an iterative method, resulting in an overall inner–outer iterative scheme for the solution of (2). Since the regularization parameter λ needs to be chosen, we adopt a so-called hybrid method [5,6], which typically projects the original problem (1) onto Krylov subspaces of increasing dimension and applies regularization to the projected problem, allowing for an efficient, adaptive (iteration-dependent) choice of λ. More details on the projection process are given in the next paragraph.

For particular instances of (2), e.g., for standard Tikhonov with $\Omega \left(x\right)={∥x∥}_{2}^{2}$, first projecting (1) and then applying standard Tikhonov to the projected problem is equivalent to first applying standard Tikhonov (2) and then projecting the regularized problem; see [1] (Chapter 6). The application of hybrid methods to Tikhonov regularized problems in general form, such as the ones in the sequence (4), is usually not straightforward and one possible approach is to first perform a transformation into standard Tikhonov form, and then apply a hybrid method to the transformed problem; see, e.g., [1,33]. In the following, we will tailor our discussions to the case $\Omega \left(x\right)=∥W_{k}D_{2d}{x∥}_{2}^{2}$, where $W_{k}=W_{\mathrm{TV}}\left(x_{k-1,★}\right)$ as in (8), $W_{k}=W_{\mathrm{aTV}}\left(x_{k-1,★}\right)$ as in (10), or $W_{k}=W_{k}^{\mathrm{diag}}$ as in (11). We remark that, since all these diagonal weighting matrices are nonsingular, the null spaces of both $W_{k}D_{2d}$ and $D_{2d}$ are spanned by the constant vectors, i.e., multiples of 1. Problem (4) specifically formulated for these cases reads

(12) $$x_{k,★}=\arg\min_{x\in R^{n}}{∥Ax-b∥}_{2}^{2}+\lambda {∥W_{k}D_{2d}x∥}_{2}^{2}\;,\;k=1,2,\ldots .$$

Let us assume that the null spaces of $A\in R^{m\times n}$ and $W_{k}D_{2d}\in R^{2\tilde{n}\times n}$ intersect trivially; this is a reasonable assumption, as the null space of *A* is typically spanned by highly oscillatory vectors; see [1] (Chapter 2). Then, problem (12) has a unique solution $x_{k,*}$, which can be equivalently expressed by computing

(13) $${\bar{y}}_{k,★}=\arg\min_{\bar{y}\in R^{2\tilde{n}}}∥\bar{A}\bar{y}-\bar{b}{∥}_{2}^{2}+\lambda {∥\bar{y}∥}_{2}^{2}\;,\;\mathrm{where}\;\begin{array}{ccc} \bar{A} & = & A{\left(W_{k}D_{2d}\right)}_{A}^{†}\\ \bar{b} & = & b-Ax_{0}\\ x_{k,★} & = & {\left(W_{k}D_{2d}\right)}_{A}^{†}{\bar{y}}_{k,★}+x_{0} \end{array}\;.$$

In the above formulation, the matrix ${\left(W_{k}D_{2d}\right)}_{A}^{†}$ is the so-called *A*-weighted pseudoinverse of $W_{k}D_{2d}$, and $x_{k,★}$ is expressed as the sum of two components: the first term belongs to the range of ${\left(W_{k}D_{2d}\right)}_{A}^{†}$, while the second term $x_{0}$ belongs to the null space of $W_{k}D_{2d}$. We refer to [26,34] for detailed derivations. In practice, following [33] and letting $K={\left(n\right)}^{-1/2}\;1\in R^{n\times 1}$ be the ‘matrix’ whose orthonormal column spans the null space of $W_{k}D_{2d}$, we can rewrite

(14) $${\left(W_{k}D_{2d}\right)}_{A}^{†}=E{\left(W_{k}D_{2d}\right)}^{†}\in R^{n\times 2\tilde{n}},\;\mathrm{where}\;E=\left(I-K{\left(AK\right)}^{†}A\right)\in R^{n\times n}$$

and ${\left(W_{k}D_{2d}\right)}^{†}$ is the Moore–Penrose pseudoinverse of $\left(W_{k}D_{2d}\right)$. We also have

(15) $$x_{0}={\left(A\left(I-{\left(W_{k}D_{2d}\right)}^{†}\left(W_{k}D_{2d}\right)\right)\right)}^{†}b=K{\left(AK\right)}^{†}b\;.$$

Computing $x_{0}$ as in (15) and performing matrix–vector products with the matrix *E* defined in (14) is computationally very cheap. Indeed, by letting $v=AK\in R^{m}$, it follows that

(16) $$v^{†}=v^{T}/{∥v∥}_{2}^{2}\;\mathrm{and}\;K{\left(AK\right)}^{†}={\left(n\right)}^{-1/2}{∥v∥}_{2}^{-2}\;1\;v^{T}\;.$$

Computing ${\left(W_{k}D_{2d}\right)}^{†}$ is nontrivial, and strategies to deal with this are explained in Section 4.

We now describe how problem (13) can be efficiently solved via a Krylov projection method based on the Golub–Kahan bidiagonalization (GKB) algorithm [35] applied to $\bar{A}$ and $\bar{b}$, whose *i*th iteration updates partial factorizations of the form

(17) $$\begin{array}{c} \bar{A}V_{i}^{\mathrm{GKB}}=U_{i+1}^{\mathrm{GKB}}{\hat{B}}_{i}^{\mathrm{GKB}}\;,\;{\bar{A}}^{T}U_{i+1}^{\mathrm{GKB}}=V_{i+1}^{\mathrm{GKB}}{\left(B_{i+1}^{\mathrm{GKB}}\right)}^{T}\;, \end{array}$$

where $V_{i}^{\mathrm{GKB}}=\left[v_{1}^{\mathrm{GKB}},\ldots ,v_{i}^{\mathrm{GKB}}\right]\in R^{n\times i}$ and $U_{i+1}^{\mathrm{GKB}}=\left[u_{1}^{\mathrm{GKB}},\ldots ,u_{i+1}^{\mathrm{GKB}}\right]\in R^{m\times (i+1)}$, with $u_{1}^{\mathrm{GKB}}=\bar{b}/{∥\bar{b}∥}_{2}$, are matrices whose orthonormal columns span the Krylov subspaces $K_{i}\left({\bar{A}}^{T}\bar{A},{\bar{A}}^{T}\bar{b}\right)$ and $K_{i}\left(\bar{A}{\bar{A}}^{T},\bar{b}\right)$, respectively; ${\hat{B}}_{i}^{\mathrm{GKB}}\in R^{(i+1)\times i}$ and $B_{i+1}^{\mathrm{GKB}}\in R^{\left(i+1)\times (i+1\right)}$ are lower bidiagonal matrices, and ${\hat{B}}_{i}^{\mathrm{GKB}}$ coincides with $B_{i+1}^{\mathrm{GKB}}$ without its last column. We refer to [1] (§ 6.3) for more details. The cost of updating factorizations (17) is dominated by four matrix–vector products (namely, with *A*, $A^{T}$, ${\left(W_{k}D_{2d}\right)}_{A}^{†}$, ${\left({\left(W_{k}D_{2d}\right)}_{A}^{†}\right)}^{T}$) at each iteration. We impose that the *i*th approximation ${\bar{y}}_{k,★}^{\left(i\right)}$ of ${\bar{y}}_{k,★}$ belongs to the space $K_{i}\left({\bar{A}}^{T}\bar{A},{\bar{A}}^{T}\bar{b}\right)$, i.e., we compute

(18) $${\bar{y}}_{k,★}^{\left(i\right)}=V_{i}^{\mathrm{GKB}}s_{i},\;\mathrm{where}\;s_{i}=\arg\min_{s\in R^{i}}∥{\hat{B}}_{i}^{\mathrm{GKB}}s-∥\bar{b}{∥}_{2}e_{1}{∥}_{2}^{2}+\lambda {∥s∥}_{2}^{2}$$

and where $e_{1}$ denotes the first canonical basis vector of $R^{\left(i+1\right)}$. The projected Tikhonov problem in (18) is of size O(i) and it is obtained by exploiting decomposition (17) and the properties of the matrices appearing therein. The regularization parameter λ can be efficiently set at each iteration, using well-known parameter choice strategies; see, e.g., [6] (§ 3). The corresponding *i*th approximation $x_{k,★}^{\left(i\right)}$ to problem (12) is computed by taking

(19) $$\begin{array}{cc} x_{k,★}^{\left(i\right)} & \;\;=\;x_{0}+{\left(W_{k}D_{2d}\right)}_{A}^{†}{\bar{y}}_{k,★}^{\left(i\right)}=x_{0}+{\left(W_{k}D_{2d}\right)}_{A}^{†}V_{i}^{\mathrm{GKB}}s_{i}\\ \; & \;\;\;\in \;x_{0}\;+\;K_{i}\left({\left(W_{k}D_{2d}\right)}_{A}^{†}{\left({\left(W_{k}D_{2d}\right)}_{A}^{†}\right)}^{T}A^{T}A,{\left(W_{k}D_{2d}\right)}_{A}^{†}{\left({\left(W_{k}D_{2d}\right)}_{A}^{†}\right)}^{T}A^{T}\left(b-Ax_{0}\right)\right)\;. \end{array}$$

We see that $x_{k,★}^{\left(i\right)}$ defined above is computed by a hybrid projection method applied to problem (12), after transformation into standard form; we refer to [6] for more details. We remark that, when a Krylov projection method (and, in particular, a GKB-based method) is applied to approximate the solution to (13), the regularization matrix $W_{k}D_{2d}$ affects the approximation subspace for the solution (typically improving it), and ${\left(W_{k}D_{2d}\right)}_{A}^{†}$ can be formally regarded as an appropriate preconditioner for the linear system in (1) (although usually it does not speed up the convergence of the Krylov solver); we refer to [1,33] (Chapter 8) for more details.

Although hybrid projection methods applied to (12) can, in general, be very efficient (meaning that, for each k=1,2,… a suitable approximation $x_{k,★}^{\left(i\right)}$ of $x_{k,★}$ in (19) is computed for i≪min{m,n}), they are still employed within an inner–outer iterative scheme that can become computationally demanding; see the results of the numerical tests reported in Section 5. In particular, the approximation subspace (19) for the solution of the *k*th problem is discarded when solving the (k+1)th problem in the sequence (12). Hybrid flexible Krylov methods have been introduced to bypass the inner–outer iterative scheme associated with (12) and generate only one solution subspace for approximating $x_{\mathrm{true}}$ by updating the weights as soon as a new approximation is computed. To apply flexible Krylov projection methods, problem (12) must first be transformed into standard form (13), so that the interpretation of ${\left(W_{k}D_{2d}\right)}_{A}^{†}$ as a preconditioner can be exploited. In general, the *i*th iteration of a hybrid flexible Krylov method computes

(20) $${\bar{x}}^{\left(i\right)}=\arg\min_{\bar{x}\in Z_{i}}∥A\bar{x}{-b∥}_{2}^{2}+\lambda {∥M_{i}\bar{x}∥}_{2}^{2}\;,\;i=1,2,\ldots ,\;x^{\left(i\right)}={\bar{x}}^{\left(i\right)}+x_{0}\;,$$

where the approximation subspace $Z_{i}$ has dimension *i* and depends on $W_{j}=W\left(D_{2d}x^{\left(j-1\right)}\right)$, j=1,…,i, and where different (more or less theoretically motivated) choices for an iteration-dependent regularization matrix $M_{i}\in R^{p_{i}\times n}$ are possible; see [17,24,25] for more details. Here, we focus on hybrid methods based on the flexible Golub–Kahan (FGK) decomposition [24], whose *i*th iteration updates partial factorizations of the form

(21) $$AZ_{i}=U_{i+1}H_{i}\;\mathrm{and}\;A^{T}U_{i+1}=V_{i+1}T_{i+1}.$$

Here, $H_{i}\in R^{(i+1)\times i}$ is upper Hessenberg, $T_{i+1}\in R^{\left(i+1)\times (i+1\right)}$ is upper triangular, and $U_{i+1}\in R^{m\times (i+1)}$ and $V_{i+1}\in R^{n\times (i+1)}$ have orthonormal columns, with

(22) $$Z_{i}=\left[{\left(W_{1}D_{2d}\right)}_{A}^{†}{\left({\left(W_{1}D_{2d}\right)}_{A}^{†}\right)}^{T}v_{1},\ldots ,{\left(W_{i}D_{2d}\right)}_{A}^{†}{\left({\left(W_{i}D_{2d}\right)}_{A}^{†}\right)}^{T}v_{i}\right]\in R^{n\times i}.$$

Similar to standard preconditioned GKB (17), the cost of updating factorizations (21) at the *i*th iteration, i=1,2,…, is dominated by four matrix–vector products (namely, with *A*, $A^{T}$, ${\left(W_{i}D_{2d}\right)}_{A}^{†}$, ${\left({\left(W_{i}D_{2d}\right)}_{A}^{†}\right)}^{T}$). However, differently from GKB-based methods, the approximation subspace $Z_{i}$, spanned by the columns of $Z_{i}$, is no longer a Krylov subspace. Despite these differences, the FGK-based projected problem associated with (20) computes

(23) $${\bar{x}}^{\left(i\right)}=Z_{i}s_{i}\in Z_{i},\;\mathrm{where}\;s_{i}=\underset{s\in R^{i}}{arg min}∥{H_{i}s-{∥\bar{b}∥}_{2}e_{1}∥}_{2}^{2}+\lambda ∥{N_{i}s∥}_{2}^{2},\;x^{\left(i\right)}={\bar{x}}^{\left(i\right)}+x_{0}\;,$$

where $N_{i}$ is a projected version of the regularization matrix $M_{i}$ appearing in (20); see also (25) for more details. Problem (23) is formally similar to (18) and (19). As in (18), allowing some heuristics, the regularization parameter λ in (23) can be efficiently set at each iteration. We conclude this section by remarking that formulations (18) and (23) can be regarded as regularized LSQR and FLSQR solvers applied to (1), respectively, and, even if not considered in this paper, other regularization methods based the on the GKB and FGK algorithms are possible. For instance, one may adopt strategies linked to LSMR and FLSMR, which result in formulations similar to (18) and (23), respectively, and which have also been proven successful for large-scale problems; see [24,36].

## 4. Edge-Preserving Hybrid FGK-Based Solvers

In this section, we present more details about the new edge-preserving hybrid FGK-based solvers: we first introduce the regularization matrices $M_{i}$ and $N_{i}$ to be employed in (20) and (23), respectively, and we analyze the convergence of the new solvers in the isotropic and anisotropic total variation cases, as introduced in Section 2. We then present some implementation details, mainly dealing with the computation of pseudoinverses ${\left(W_{k}D_{2d}\right)}^{†}$.

### 4.1. Problem Setup and Convergence Analysis

Following the arguments originally presented in [17] for a simpler FGK-based solver for $\ell_{p}$ regularization, to derive formulation (20), we should start by establishing links to the GKB-based solver (18) applied to the transformed problem (13), whose approximate solution needs to be further manipulated (i.e., multiplied by ${\left(W_{k}D_{2d}\right)}_{A}^{†}$ and added to $x_{0}$) to approximate the solution to the original problem (12). In contrast, the approximate solution obtained by solving (20) only needs to be added to $x_{0}$ to approximate the solution to the original problem (12). Furthermore, if we assume that $W_{j}=W_{k}$, j=1,…,i in (22), then the space $Z_{i}$ spanned by the columns of $Z_{i}$ coincides with the Krylov subspace (19) for $x_{k,★}^{\left(i\right)}$. Therefore, the regularization term considered in (20) should regularize the solution (12), i.e., (20) should be formulated as

(24) $${\bar{x}}^{\left(i\right)}=\arg\min_{\bar{x}\in Z_{i}}∥A\bar{x}{-b∥}_{2}^{2}+\lambda {∥W_{i}D_{2d}\bar{x}∥}_{2}^{2}\;,\;i=1,2,\ldots ,\;x^{\left(i\right)}={\bar{x}}^{\left(i\right)}+x_{0}\;.$$

Correspondingly, substituting $\bar{x}=Z_{i}s$ in (24), we obtain that the regularization term to be used in the projected problem (23) has the form

(25) $$∥N_{i}{s∥}_{2}^{2}=∥W_{i}D_{2d}Z_{i}{s∥}_{2}^{2}=∥Q_{i}R_{i}{s∥}_{2}^{2}={∥R_{i}s∥}_{2}^{2},\;\mathrm{where}\;W_{i}D_{2d}Z_{i}=Q_{i}R_{i}$$

which is the economy-size QR factorization of $W_{i}D_{2d}Z_{i}$. The cost of computing $R_{i}$ is of order $O(\tilde{n}i^{2})$ and, therefore, it is negligible when i≪min{n,m}. The above derivations ensure that problem (23), with the regularizer set as in (25), can be regarded as a projection of the *i*th full-dimensional reweighted Tikhonov problem (i.e., (12) with k=i). In other words, we are adopting a “first-regularize-then-project” framework (see [1] (Chapter 6)): this remark is pivotal when proving convergence results, which we present next.

Let us fix a point $x_{l}\in R^{n}$ and define the quadratic functional of *x*

$$Q\left(x;x_{l}\right)={∥Ax-b∥}_{2}^{2}+\lambda \left(∥W_{l}D_{2d}{x∥}_{2}^{2}+\Omega \left(x_{l}\right)\right)\;,$$

where

(26) $$\begin{array}{cccc} W_{l}=W_{\mathrm{TV}}\left(D_{2d}x_{l}\right) & \mathrm{and} & \Omega \left(x\right)=∥W_{\mathrm{TV}}\left(D_{2d}x\right)D_{2d}{x∥}_{2}^{2} & \mathrm{as}\;\mathrm{in}\;(7),\\ W_{l}=W_{\mathrm{aTV}}\left(D_{2d}x_{l}\right) & \mathrm{and} & \Omega \left(x\right)=∥W_{\mathrm{aTV}}\left(D_{2d}x\right)D_{2d}{x∥}_{2}^{2} & \mathrm{as}\;\mathrm{in}\;(9), \end{array}$$

for the (smoothed) TV(x) and aTV(x) cases, respectively. Recalling that $\hat{\lambda }=2\lambda$, it is immediate that

$$Q\left(x_{l};x_{l}\right)={∥Ax_{l}-b∥}_{2}^{2}+\hat{\lambda }\Omega \left(x_{l}\right)=:F\left(x_{l}\right),$$

so that $Q(x;x_{l})$ is tangent in $x_{l}$ to the objective function F(x) in (2). It is also possible to prove that

$$\nabla Q\left(x_{l};x_{l}\right)=\nabla F\left(x_{l}\right)\;\mathrm{and}\;F\left(x\right)\leq Q\left(x;x_{l}\right)\;\mathrm{for}\;\mathrm{all}\;x\in R^{n}\;,$$

so that $Q(x;x_{l})$ is a quadratic tangent majorant in $x_{l}$ to the original function F(x), where Ω(x) is chosen as in (26). We refer to [16] for full derivations in the TV(x) case, which also hold for the simpler aTV(x) case; see, for instance, [19,23].

Now, consider the sequence ${\left\{Q\left(x^{\left(i\right)};x^{\left(i\right)}\right)\right\}}_{i\geq 1}$, where $x^{\left(i\right)}$ is the solution of the *i*th hybrid FGK problem (23), with $∥N_{i}{x∥}_{2}^{2}$ chosen as in (25): one can prove that such a sequence is monotonically decreasing and that it is bounded below by zero, using the same arguments as in [17] (Lemma 3.3). As a consequence, such a sequence has a stationary point and, using the same arguments as in [19] (Theorem 5), it can be proven that $\lim_{i\to \infty }{∥x^{\left(i\right)}-x^{\left(i-1\right)}∥}_{2}=0$ and ${\left\{x^{\left(i\right)}\right\}}_{i\geq 1}$ converges to the unique solution of (2), with Ω(x) chosen as in (26).

Although it is clear that the arguments presented in this section only hold when the regularization parameter λ is fixed, an iteration-dependent choice of λ can be naturally and heuristically implemented within the new FGK-based solvers (see Section 4.3 for a possible strategy), following the common practice established for other hybrid Krylov projection methods [6,18]. Numerical experimentation shows that the new solvers are robust with respect to adaptive parameter choice; see Section 5 for more details.

### 4.2. Standard Form Transformation Computations

As already hinted in Section 3, the cost of each iteration of new edge-enhancing hybrid FGK-based solvers is dominated by matrix–vector products with *A*, $A^{T}$, ${\left(W_{k}D_{2d}\right)}_{A}^{†}$, and ${\left({\left(W_{k}D_{2d}\right)}_{A}^{†}\right)}^{T}$; the latter are dominated by the cost of performing matrix–vector products with ${\left(W_{k}D_{2d}\right)}^{†}$ and ${\left({\left(W_{k}D_{2d}\right)}^{†}\right)}^{T}$, respectively (see Equations (14)–(16)). Unfortunately, computing ${\left(W_{k}D_{2d}\right)}^{†}$ is not straightforward. Indeed, as already highlighted in [30], it is not possible to exploit the structure of $W_{k}D_{2d}$ for efficient computations: this would be possible if only the $D_{2d}$ term was considered but, because $D_{2d}$ is overdetermined,

(27) $${\left(W_{k}D_{2d}\right)}^{†}\neq D_{2d}^{†}W_{k}^{-1}=:{\tilde{L}}^{†}\;.$$

As suggested in [30], to keep the computations cheap, we run the hybrid FGK solver by performing matrix–vector products with the approximation ${\tilde{L}}^{†}$ of ${\left(W_{k}D_{2d}\right)}^{†}$, and with ${\left({\tilde{L}}^{†}\right)}^{T}$. As shown below, ${\tilde{L}}^{†}$ can be regarded as the pseudoinverse of $W_{k}D_{2d}$ computed in the $W_{k}^{-2}$ norm. Namely, recalling the characterization

(28) $${\left(W_{k}D_{2d}\right)}^{†}s=\arg\min_{t\in R^{n}}{∥(W_{k}D_{2d})t-s∥}_{2},$$

we have that

$$\begin{array}{ccc} {\tilde{L}}^{†}s=\arg\min_{t\in R^{n}}{∥D_{2d}t-W_{k}^{-1}s∥}_{2} & = & \arg\min_{t\in R^{n}}{∥W_{k}D_{2d}t-s∥}_{W_{k}^{-2}}\\ & = & {\left(W_{k}D_{2d}t-s\right)}^{T}W_{k}^{-2}\left(W_{k}D_{2d}t-s\right)\;. \end{array}$$

Following the derivations in [33], once the singular value decomposition (SVD) of $D_{1d}\in R^{(N-1)\times N}$, namely $D_{1d}=U_{1d}\Sigma_{1d}V_{1d}^{T}$, is computed, matrix–vector products with $D_{2d}^{†}$ and ${\left(D_{2d}^{†}\right)}^{T}$ can be performed by first computing the SVD of $D_{2d}$ as follows:

$$\begin{array}{cc} D_{2d} & =\left[\begin{array}{c} D_{1d}⊗\;I\\ I⊗D_{1d} \end{array}\right]=\left[\begin{array}{cc} U_{1d}⊗V_{1d} & 0\\ 0 & V_{1d}⊗U_{1d} \end{array}\right]{\tilde{Q}}^{T}\tilde{D}{\left[\begin{array}{c} V_{1d}⊗V_{1d} \end{array}\right]}^{T},\\ \; & \;\mathrm{where}\;\tilde{Q}\tilde{D}=\tilde{\Sigma }=\left[\begin{array}{c} \Sigma_{1d}⊗\;I\\ I⊗\Sigma_{1d} \end{array}\right]\;, \end{array}$$

and where $\tilde{Q}\in R^{2\tilde{n}\times 2\tilde{n}}$ is an orthogonal matrix implicitly obtained by applying a set of Givens rotations to the sparse matrix $\tilde{\Sigma }$, and $\tilde{D}\in R^{2\tilde{n}\times n}$ is a nonnegative diagonal matrix of rank n−1. By using standard properties of pseudoinverses,

$$D_{2d}^{†}=\left[\begin{array}{c} V_{1d}⊗V_{1d} \end{array}\right]{\tilde{D}}^{†}\tilde{Q}\left[\begin{array}{cc} U_{1d}^{T}⊗V_{1d}^{T} & 0\\ 0 & V_{1d}^{T}⊗U_{1d}^{T} \end{array}\right]\;.$$

The procedure outlined above to compute ${\tilde{L}}^{†}$ costs $O(n^{3/2})$ flops. Alternatively, as suggested in [30], one can employ a (preconditioned) iterative method to solve the least squares problem (28).

Inverting the nonsingular diagonal weighting matrices as in (27) is straightforward and costs O(n) flops. However, when considering the weights (11), the results obtained by computing ${\left(W_{k}^{\mathrm{diag}}\right)}^{-1}=\mathrm{diag}\left({\left(w_{k}^{\mathrm{diag}}\right)}^{-1}\right)$ are consistently poor: this may be related to the fact that $w_{k}^{\mathrm{diag}}$ is expressed as the product of *k* diagonal matrices defined with respect to all the previous approximate solutions. To circumvent this problem, we consider the first-order approximation of the entries of ${\left(W_{k}^{\mathrm{diag}}\right)}^{-1}$ around 0, and take

$${\left(W_{k}^{\mathrm{diag}}\right)}^{-1}≃\mathrm{diag}\left(1+w_{k}^{\mathrm{diag}}\right)\;.$$

We remark that all the weights (8), (10) and (11) are dependent on the latest available approximate solution and on τ; the latter is fixed for all the iterations and does not have an impact on the computed solutions, provided that its value is reasonably small. In our implementation, $\tau =10^{-10}$; see Section 5 for more details. Finally, we note that the quantities $x_{0}$ and *E* defined in (14)–(16) are independent of the weighting matrix $W_{k}$: they are easy to compute and this can be done ahead of the iterations.

### 4.3. Choosing λ and Stopping the Iterations

The new hybrid FGK-based solvers, as with all the hybrid solvers, are effective only when the regularization parameter λ and a stopping criterion for the iterations are properly chosen; see, e.g., [6] (§ 3). The regularization parameter λ can be adaptively and automatically set at each iteration, i.e., a value $\lambda =\lambda_{i}$ can be heuristically set for the *i*th projected problem (23). Strategies to set $\lambda_{i}$ are well-established, and can often be regarded as the projected versions of popular parameter choice rules for 2-norm Tikhonov regularized problems similar to (12). The upside of applying these strategies to the projected problem (23) is that, at the *i*th iteration, only computations with matrices of size O(i) need to be performed, which results in negligible computational overhead when i≪min{m,n}; see [17,18,23]. To better highlight the dependence of the computed solution $x^{\left(i\right)}$ and $s_{i}$ on λ, in this section, we use the notation $x^{\left(i\right)}\left(\lambda \right)$ and $s_{i}\left(\lambda \right)$, respectively.

Assuming that a good approximation of the 2-norm of the noise vector *e* appearing in (1) is available, at each iteration, we apply the discrepancy principle, i.e., we compute $\lambda =\lambda_{i}$ such that

(29) $$∥b-Ax^{\left(i\right)}{\left(\lambda \right)∥}_{2}=\eta {∥e∥}_{2}\;,$$

where η is a user-specified ‘safety’ factor (typically slightly larger than 1) that prevents overfitting the noise. The equation above is guaranteed to have a solution as soon as $∥b-Ax^{\left(i\right)}{\left(0\right)∥}_{2}\leq {∥e∥}_{2}$, which is typically the case after a few FGK iterations. Under this assumption, a zero finder is applied to approximate $\lambda_{i}$ working with the projected quantities, since, from (21) and (23),

(30) $$∥b-Ax^{\left(i\right)}{\left(\lambda \right)∥}_{2}=∥b-A\left(x_{0}+Z_{i}s_{i}\left(\lambda \right)\right){∥}_{2}=∥∥\bar{b}{{∥}_{2}e_{1}-H_{i}s_{i}\left(\lambda \right)∥}_{2}\;.$$

If ${∥e∥}_{2}$ is overestimated (resp. underestimated), the solution $x^{\left(i\right)}\left(\lambda \right)$ satisfying (29) is typically over-regularized (resp. under-regularized); we refer to [1] (Chapter 5) for more details. An estimate of ${∥e∥}_{2}$ may be obtained from, e.g., the highest coefficients of the noisy data under some transformation, such as wavelets; see [37]. If a good estimate of ${∥e∥}_{2}$ is not available, alternative parameter choice strategies that do not require this quantity can be used; see, e.g., [6].

We stop the FGK iterations when some stabilization of the regularization parameter is detected, i.e., at the first iteration *k* such that

(31) $$\frac{|\lambda_{k}-\lambda_{k-1}|}{\lambda_{k}}<\xi \;and\;\frac{|\lambda_{k-1}-\lambda_{k-2}|}{\lambda_{k-1}}<\xi \;,$$

where k>2 and ξ>0 is a user-specified threshold. We also stress that, if a suitable value of $\lambda_{i}$ is set at each iteration, the quality of the reconstructions computed as the iterations proceed does not significantly deteriorate.

The main steps of the new FGK-based methods are summarized in Algorithm 1.
**Algorithm 1:** Edge-enhancing FGK-based methods.1:**Input**: initial guess $x_{0,★}$, $r_{0,★}=b-Ax_{0,★}$, thresholds τ,η,ξ>0,2:Take $u_{1}=r_{0,★}/∥r_{0,★}∥$, $U_{1}=\left[u_{1}\right]$, $V_{0}=\left[\right]$, $Z_{0}=\left[\right]$3:Compute $x_{0}$ and *E* as in (14)–(16)4:**for**i=1,2,… until (31) is satisfied **do**5: Expand the approximation subspace, by updating the FGK factorization (21)
$$\;\begin{array}{ccc} {\bar{v}}_{i}=A^{T}u_{i},\; & v=\left(I-V_{i-1}V_{i-1}^{T}\right){\bar{v}}_{i},\;v_{i}=v/{∥v∥}_{2},\; & V_{i}=\left[V_{i-1},v_{i}\right]\\ z_{i}={\left(W_{i}D_{2d}\right)}^{†}v_{i}\; & (using()),\; & Z_{i}=\left[Z_{i-1},z_{i}\right]\\ {\bar{u}}_{i+1}=Az_{i},\; & u=\left(I-U_{i}U_{i}^{T}\right){\bar{u}}_{i+1},\;u_{i+1}=u/{∥u∥}_{2},\; & U_{i+1}=\left[U_{i},u_{i+1}\right] \end{array}$$6: Choose $\lambda =\lambda_{i}$ such that (30) holds7: Solve problem (24) with $\lambda =\lambda_{i}$8: Update $W_{i+1}$9:**end for**

## 5. Numerical Experiments

In this section, we present the results of some numerical experiments that demonstrate the performance of the new edge-preserving FGK-based solvers, applied with the regularization terms and weightings described in Section 2. We consider test problems modelling image deblurring of a piecewise constant image with low TV, inpainting combined with image deblurring for an image with high TV, and an undersampled computed tomography problem. To validate the new methods, we provide comparisons with the IRN methods [16] equipped with the same regularization terms as the FGK-based methods, the GKS method for TV [19,23], a forward–backward solver for TV (FBTV) [13], and an LSQR-based hybrid Krylov method for (2), with $\Omega \left(x\right)=∥D_{2d}{x∥}_{2}^{2}$ [6]. The acronyms used to denote the different methods considered in this section, as well as a common color code employed in most figures, are summarized in Table 1. When comparing with IRN (4), we take $W_{1}$ as the identity matrix, $L=D_{2d}$, and compute each $x_{k,★}$, k=1,2,… using an LSQR-based hybrid Krylov method. When considering weights, the value $\tau =10^{-10}$ is chosen as a smoothing parameter in (5) and (11). When running the new FGK-based solvers, as well as the IRN, GKS, and LSQR-based hybrid methods, λ is chosen at each iteration according to the discrepancy principle (29), with η=1.01; the stopping criterion is given by (31), with ξ=0.9 (note that, in the IRN case, this holds only for the inner IRN iterations). The (fixed) regularization parameter used by the FBTV method is chosen for all the experiments as $\lambda =\lambda_{FB}=2\times 10^{-3}\;∥{D_{2d}A^{T}b∥}_{\infty }$, which is found experimentally to perform well; FBTV also requires the choice of a step-size $\tau_{FB}$, which is determined either according to the forward–backward theory (i.e., $\tau_{FB}=\sigma_{1}{\left(A\right)}^{-2}$, where $\sigma_{1}\left(A\right)$ is the largest singular value of *A* approximated by running a few LSQR iterations) or in an optimal way (i.e., by initially running 30 iterations of the FBTV method for different values of $\tau_{FB}$ logarithmically equispaced between $10^{-5}$ and $10^{2}$, and then selecting the parameter $\tau_{FB}$ that realizes the smallest residual norm). Unless otherwise stated, all algorithms run for 200 iterations, even if the stopping criterion (31) is satisfied, to observe their long-term behavior; we indicate the iteration that satisfies the stopping criterion with a diamond marker in the relevant plots. In all experiments, the quality of the reconstructions is measured by both the relative restoration error (RRE) $∥{x^{\left(k\right)}-x_{{}_{\mathrm{true}}}∥}_{2}/∥{x_{{}_{\mathrm{true}}}∥}_{2}$ and the structure similarity (SSIM) index between $x^{\left(k\right)}$ and $x_{{}_{\mathrm{true}}}$ [38], where $x^{\left(k\right)}$ is the solution computed at the *k*th iteration of each solver. All experiments were performed in MATLAB R2020a and utilized functions from the IR Tools [39] package; our codes are publicly available (MATLAB functions implementing the new FGK-based edge enhancing solvers and some test scripts are available on github, https://github.com/silviagazzola/EdgeEnhancingFGK, accessed on 25 September 2021).

### 5.1. Experiment 1—Image Deblurring

The first experiment involves deblurring a piecewise constant test image displaying geometric patterns. We consider two versions of the test image, one of size 32×32 pixels and one of size 256×256 pixels. The images are corrupted by Gaussian blur and additive Gaussian white noise *e* with relative noise level ${∥e∥}_{2}/∥{b_{\mathrm{true}}∥}_{2}=0.01$. This test problem can be generated using the IR Tools package with

$$\begin{array}{cc} & \;\;\mathrm{PbOpt}=\mathrm{PRset}{(}^{\prime }{\mathrm{trueimage}}^{\prime }{,}^{\prime }\mathrm{pattern}1^{\prime }{,}^{\prime }{\mathrm{BlurLevel}}^{\prime }{,}^{\prime }{\mathrm{blevel}}^{\prime });\\ & [A,\mathrm{btrue},\mathrm{xtrue},\mathrm{ProbInfo}]=\mathrm{PRblur}(N,\mathrm{PbOpt});\\ & b=\mathrm{PRnoise}(\mathrm{btrue},1e-2); \end{array}$$

**Figure 1:**
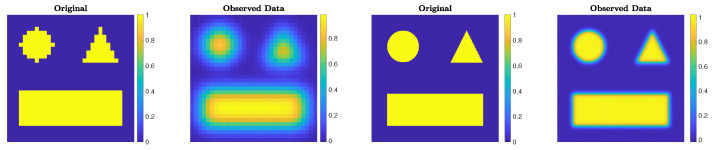
Experiment 1. Simple geometric pattern that has been blurred by some Gaussian blur and corrupted by some Gaussian noise. From the left, the first and second frames are for the N = 32 case, and the third and fourth frames are for the N = 256 case.

The choices N = 32 and blevel = ’mild’, and N = 256 and blevel = ’medium’, are made. The ground truths and the corrupted images are displayed in Figure 1.

**Figure 2:**
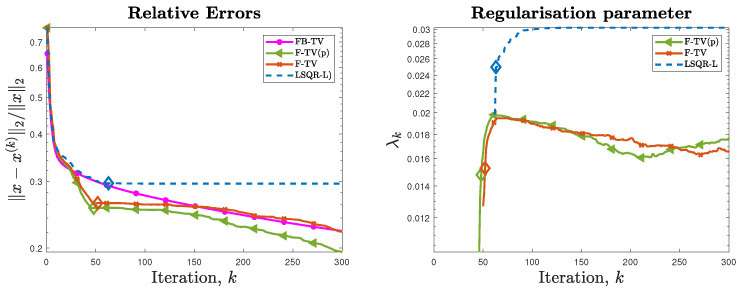
Experiment 1, with N = 32. (**Left frame**): History of the relative error norms, comparing FBTV, F-TV, F-TV(p), and LSQR-L. (**Right frame**): History of the automatically selected regularization parameter for the projected problem (for the F-TV, F-TV(p), and LSQR-L methods), according to the discrepancy principle (29). Diamond markers indicate the iteration at which the stopping criterion (31) is satisfied.

**Figure 3:**
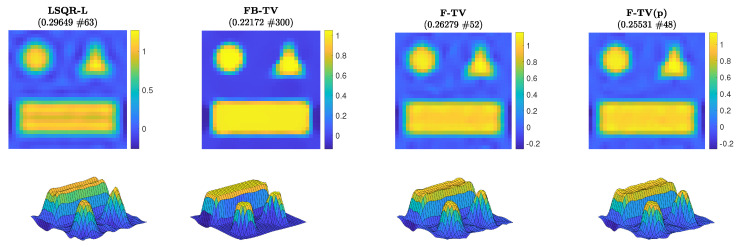
Experiment 1, with N = 32. Reconstructed solutions from LSQR-L, FBTV, F-TV, and F-TV(p) when the respective stopping criterion has been satisfied. The RRE and corresponding stopping iteration are displayed in the frame titles.

First of all, we compare the RREs achieved by different methods for the test problem of size N = 32. We run the new FGK method F-TV(p) with true pseudoinverse $L^{†}={\left(W_{k}D_{2d}\right)}^{†}$ computed directly, as well as the new F-TV method, wherein the alternative pseudoinverse ${\tilde{L}}^{†}=D^{†}W^{-1}$ is used in lieu of $L^{†}$ at each iteration of the flexible framework. We also consider FBTV, with a regularization parameter $\lambda_{FB}=0.0014$ and optimal step-size $\tau_{FB}=1.8330$. The graphs of the RREs versus iteration number are shown in Figure 2 (left frame). For this test problem, the solvers are run for 300 iterations and we can clearly see that each of the F-TV, F-TV(p), and LSQR-L methods terminate early due to the stopping criterion (31). The RREs in Figure 2 show that both F-TV and F-TV(p) follow very similar error histories, and terminate close to one another at iterations 48 and 52, respectively (highlighted by diamond markers), with an RRE around 0.25. This behavior provides experimental evidence supporting the use of the approximate pseudoinvese ${\tilde{L}}^{†}=D^{†}W^{-1}$ in the following test problems. FBTV sees steady improvement of the RRE over all iterations, and achieves an RRE similar to F-TV at iteration 150. Plots of the regularization parameter automatically selected when solving the projected problem are provided in the right-hand frame of Figure 2. We see that no Tikhonov regularization is enforced in the projected problem (23) until iteration 46; however, since the projected problem (23) is associated with the standard-form transformed problem (13), even if λ=0, the regularization matrix $W_{k}D_{2d}$ affects the transformed coefficient matrix $\bar{A}$, thus influencing the solutions $x^{\left(k\right)}$ at the *k*th iteration. Although not shown in the following, the adaptively chosen regularization parameter exhibits a behavior similar to the one displayed in Figure 2, i.e., it is initially zero and then quickly stabilizes around a suitable value. The reconstructions achieved when the stopping criterion is satisfied are displayed in Figure 3; corresponding surface plots are also displayed to better indicate their ideally piecewise-constant features. The iteration number and associated RRE are also reported. Since the geometric pattern test image is piecewise constant, total variation is a suitable choice of regularizer as it promotes such a property in the reconstructions. Indeed, the reconstructions computed by FBTV, F-TV, and F-TV(p) reproduce such features, whereas LSQR-L, which uses a fixed regularization matrix $D_{2d}$, does not (e.g., it yields a reconstruction that has a distinct valley in the rectangular shape). The reconstruction from FBTV has the smallest RRE out of the presented reconstructions. However, we see in Figure 2 that, while the RREs of both F-TV and F-TV(p) temporarily stagnate after the stopping criterion is satisfied, they then continue to decrease to levels comparable with that of the presented FBTV reconstruction.

**Figure 4:**
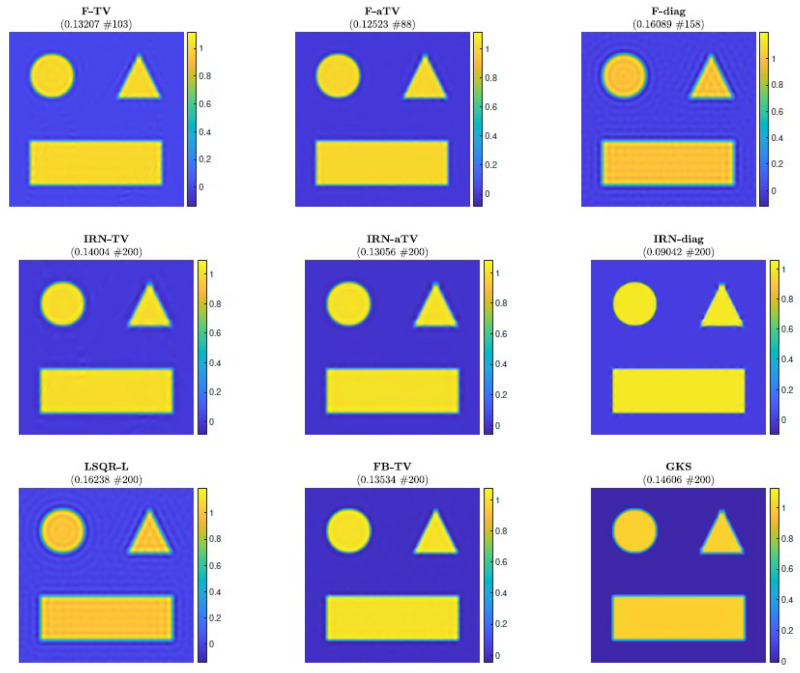
Experiment 1, with N = 256. Reconstructions achieved by various methods at the stopping iteration (if a stopping criterion is used, else the maximum iteration). The RRE and stopping iteration for each reconstruction are displayed in the frame titles.

**Figure 5:**
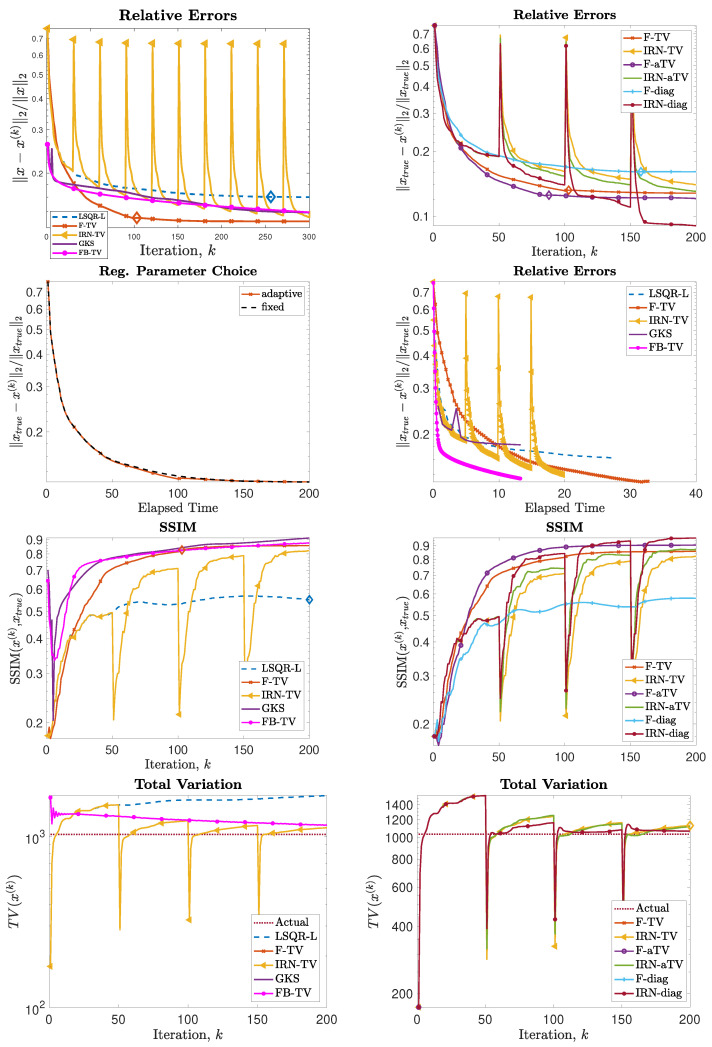
Experiment 1, with N = 256. (**Top row**): History of the relative error norms, comparing F-TV, F-aTV, F-diag, FBTV, and LSQR-L. Diamond markers indicate the iteration at which the stopping criterion (31) is satisfied. (**Second row**, **left**): history of the relative error for the new F-TV method implemented with iteration-dependent regularization parameter $\lambda^{\left(k\right)}$, k=1,2,…, set according to the discrepancy principle (30), and fixed regularization parameter $\lambda =\lambda^{\left(200\right)}$. (**Second row**, **right**): progress of the F-TV, LSQR-L, FBTV, GKS and IRN-TV relative errors versus elapsed computational time. (**Third row**): SSIMs values versus iteration number for the methods listed in Table 1. (**Bottom row**): history of the total variation of the reconstructions computed by each method.

Next, we consider the test problem of size N = 256; in this setting (as well as for all the other experiments), it is no longer feasible to compute ${\left(W_{k}D_{2d}\right)}^{†}$. Figure 4 shows the reconstructions computed by the FKS-based methods and LSQR-L at the stopping iteration, the IRN methods at the final iteration, and the FBTV and GKS methods at the iteration with the lowest RRE. We can see that both the IRN and GKS methods struggle to distinguish the corners of geometrical objects, which appear flat and truncated. GKS performs exceptionally well in recovering the correct pixel intensity scale, whereas the IRN-diag reconstruction has some pixels with intensities as low as −1 and as high as 1.8, due to spurious oscillations around the edges of the imaged objects. The reconstruction by LSQR-L has many ringing artifacts, which are removed when more sophisticated regularizers, such as isotropic total variation, are utilized. The values of the relative errors versus number of iterations for all the considered methods are plotted in Figure 5, in the upper frames. In both frames, we can clearly see that the IRN methods are affected by periodic sudden jumps in the RRE values: this is due to the fact that, in accordance with common practice (see, e.g., [40]), the IRN methods are implemented with ‘cold’ restarts, i.e., $x_{0,★}=0$ at the beginning of each iteration cycle. The frame in position (2,2) also displays the progress of the relative errors versus computational time (as measured by MATLAB’s tic toc command) for a few selected methods, where both F-TV and LSQR-L are run till the stopping criterion is statisfied and all the other solvers run for 200 iterations. We can clearly see that the running time of the new F-TV methods exceeds the running times of FISTA and GKS by approximately 20 s, and the running time of IRN-TV by approximately 10 s. However, the new F-TV method also achieves a lower RRE and it is likely that, if FISTA, GKS, and IRN-TV are run for more iterations, they will reach the same RRE, taking additional computational time. Moreover, according to the standard practice, FISTA may be run several times for different values of the regularization parameter λ before finding an appropriate one: for this numerical experiment, running FISTA for three different values of λ would be enough to exceed the F-TV computational time. The quality of the computed solutions versus the number of iterations is also displayed in the frames in the third row of Figure 5, where the SSIM is used as a quality measure. According to this metric, the reconstructions computed by F-TV, GKS, and FBTV have similar high quality, which agrees with the pictures displayed in Figure 4. As already remarked in the previous sections, one advantage of the new FGK-based solvers is that the regularization parameter λ can be adaptively and heuristically chosen at each iteration: the frame in position (2,1) of Figure 5 displays the behavior of the F-TV RRE with iteration-dependent regularization parameter $\lambda^{\left(k\right)}$, k=1,2,…, set according to the discrepancy principle (30), and fixed regularization parameter $\lambda =\lambda^{\left(200\right)}$. We can clearly see that the two approaches are almost identical, providing experimental validation for the adaptive regularization parameter choice. Although not reported, such behavior is common to F-TV and F-aTV and it is observed in all the performed numerical experiments. The history of the total variation of the reconstructions is plotted in the bottom row of Figure 5, where the total variation of the ground truth image is also included. Excluding those of IRN-diag, F-diag, and LSQR-L, the total variation of the reconstructions stabilizes at a level adherent to that of the ground truth, with the IRN-aTV, IRN-TV, and GKS reconstructions performing particularly well.

### 5.2. Experiment 2—Image Inpainting and Deblurring

**Figure 6:**
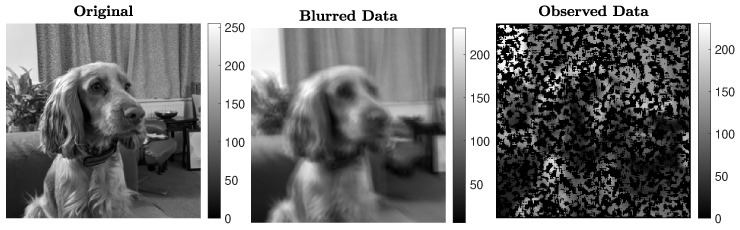
Experiment 2. Test problem involving shaking blur followed by inpainting operator.

**Figure 7:**
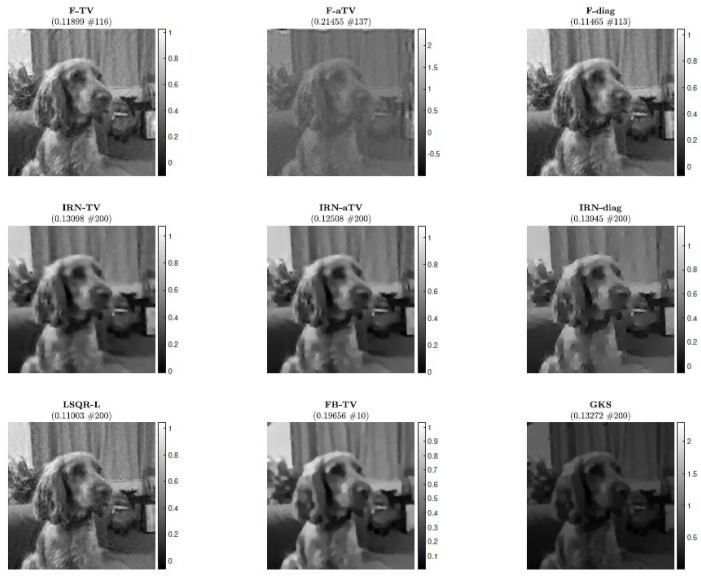
Experiment 2. Reconstructions achieved by various methods. The RRE and stopping iteration for each reconstruction are displayed in the frame title.

**Figure 8:**
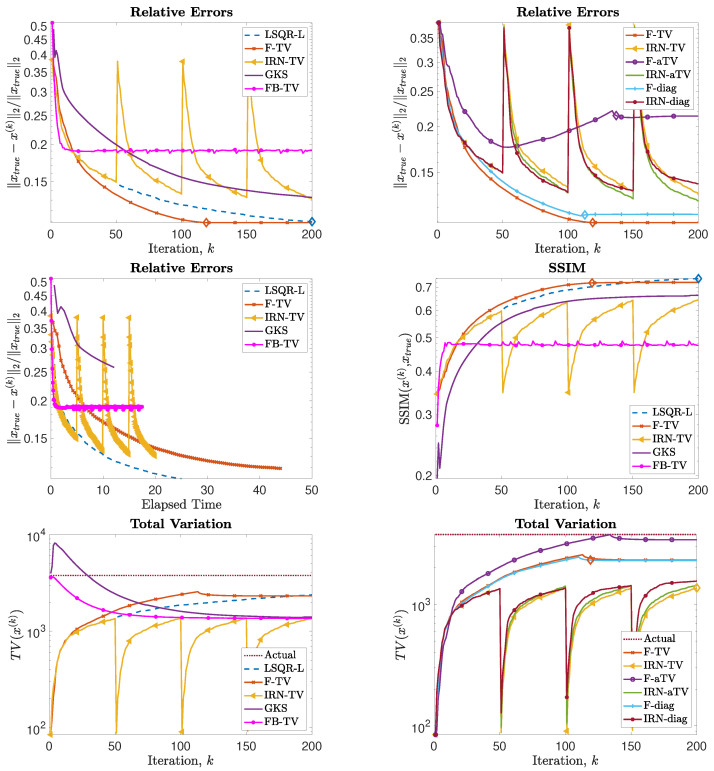
Experiment 2. (**Top row**): history of the relative error norms, comparing F-TV, F-aTV, F-diag, FBTV, and LSQR-L. Diamond markers indicate the iteration at which the stopping criterion (31) is satisfied. (**Second row**, **left**): progress of the F-TV, LSQR-L, FBTV, GKS and IRN-TV relative errors versus elapsed computational time. (**Second row**, **right**): SSIMs values versus iteration number for F-TV, LSQR-L, FBTV, GKS and IRN-TV. (**Bottom row**): history of the total variation of the reconstructions computed by each method.

In this experiment, we consider restoring a 256×256 pixel image of high total variation that has been corrupted by both blur (associated with a known blurring operator $A_{\mathrm{blur}}$) and undersampling (associated with a known operator *S*), in this order. The uncorrupted test image, blurred data, and undersampled data are shown in Figure 6. The blurring operator $A_{\mathrm{blur}}$ is generated using the following IR Tools function:

$$\mathrm{Ablur}=\mathrm{PRblurshake}{(}^{\prime }{\mathrm{CommitCrime}}^{\prime }{,}^{\prime }{\mathrm{on}}^{\prime }{,}^{\prime }{\mathrm{BlurLevel}}^{\prime }{,}^{\prime }{\mathrm{mild}}^{\prime }),$$

which models random shaking blur of mild intensity. Here, the ’CommitCrime’ option relates to whether the reflexible boundary conditions, imposed by the blurring operator, should be regarded as how the exact data precisely behave outside the frame of reference; see [39] for details. The known undersampling operator *S* picks clusters of pixels at random: approximately 40% of the pixels are retained. The forward operator associated with this test problem is therefore $A=SA_{\mathrm{blur}}$, of size 27,395×65,536. Gaussian white noise *e* of relative noise level 0.01 is added to the data. Figure 7 displays the reconstructions obtained by the considered methods, with a similar format to Figure 4. We can see that the IRN methods yield reconstructions that are more blocky and piecewise than the F-TV and the F-diag methods. LSQR-L performs well for this problem, with a reconstruction similar to those obtained by both F-TV and F-diag. This may in part be due to the original picture having piecewise constant features along with smoothly and rapidly varying features, so that penalization of the TV norm may not be as competitive an option as simply penalizing the (not necessarily sparse) gradients. We remark that it takes LSQR-L almost twice as many iterations as F-TV and F-diag to terminate via the stopping criterion (31), despite the three methods having similar RRE histories (this is visible in Figure 8). FBTV (with $\lambda_{FB}=0.5584$, $\tau_{FB}=1.8330$) attains a reconstruction of quality similar to those of IRN-TV and IRN-aTV. Both F-aTV and GKS perform poorly for this problem, with some pixels in the respective reconstruction dominating the scaling of the image and far exceeding the true pixel value range. Along with the RRE history, Figure 8 displays the SSIM history, the values of the RREs versus the elapsed computational time, and the values of the total variation of the reconstructions at each iteration.

### 5.3. Experiment 3—Computed Tomography

We consider an undersampled computed tomography test problem, with a ground truth image (phantom) of size 256×256 pixels, based on random Voronoi cells and simulating grains in a crystalline material. The forward operator represents a 2D equidistant parallel X-ray beam geometry, with data taken from angles 0 to 179 degrees in increments of 2, leading to an underdetermined matrix *A* of size 32,580×65,536. The data (sinogram) are corrupted by Gaussian white noise of level 0.01. Such a test problem can be generated from IR Tools with the following instructions:

$$\begin{array}{cc} & \;\;\mathrm{PbOpt}\;=\;\mathrm{PRset}(’\mathrm{angles}’,\;0:2:179,\;’\mathrm{phantomImage}’,\;’\mathrm{grains}’);\\ & \;[A,\;\mathrm{btrue},\;\mathrm{xtrue},\;\mathrm{ProbInfo}]\;=\;\mathrm{PRtomo}(256,\;\mathrm{PbOpt}); \end{array}$$

**Figure 9:**
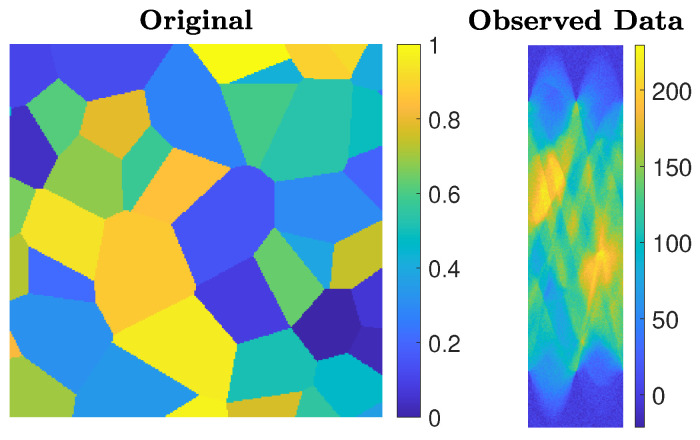
Experiment 3. Computed tomography test problem: the true object to be imaged (phantom) is displayed on the left and the collected data (sinogram) are displayed on the right.

**Figure 10:**
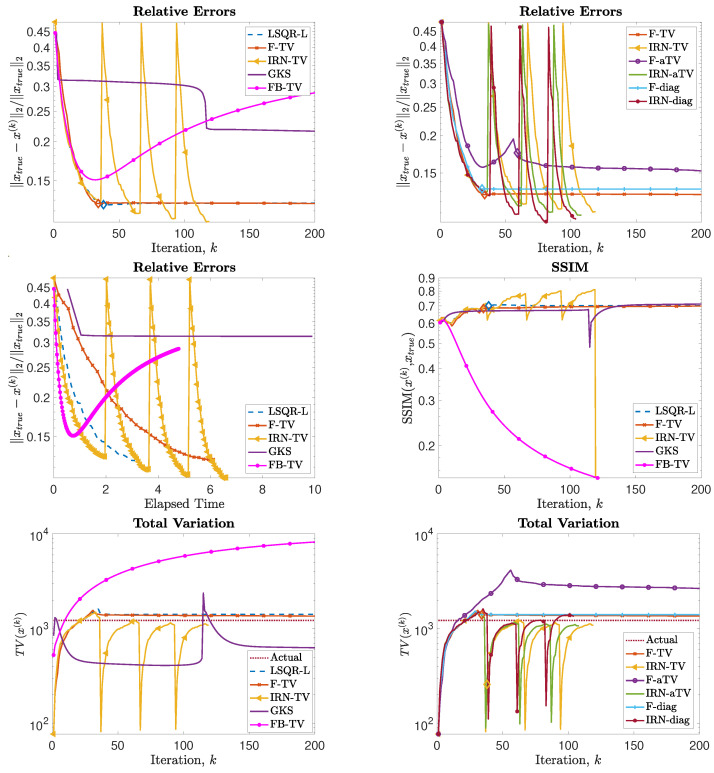
Experiment 3. (**Top row**): history of the relative error norms, comparing F-TV, F-aTV, F-diag, FBTV, and LSQR-L. Diamond markers indicate the iteration at which the stopping criterion (31) is satisfied. (**Second row**, **left**): progress of the F-TV, LSQR-L, FBTV, GKS and IRN-TV relative errors versus elapsed computational time. (**Second row**, **right**): SSIMs values versus iteration number for F-TV, LSQR-L, FBTV, GKS and IRN-TV. (**Bottom row**): history of the total variation of the reconstructions computed by each method.

The phantom and sinogram for this test problem are displayed in Figure 9. The top row of Figure 10 displays the history of RREs of the considered methods, in which not only do IRN-based methods achieve the lowest RREs, but they also terminate inner loops early—leading to around half as many overall iterations being performed. FBTV (with fixed $\lambda_{FB}=12.5881$, $\tau_{FB}=6.1585\cdot 10^{-5}$), F-TV, and LSQR-L have similar RRE histories for the first 30 iterations; however, FBTV exhibits semi-convergent properties whereas LSQR-L and F-TV stabilize due to the automatically selected regularization parameter. The GKS method exhibits inconsistent improvement of RRE between iterations, and settles at an RRE larger than the FKS and the IRN ones. The smallest RRE out of all the considered methods is achieved by IRN-diag on the third outer loop. Subsequent outer loops of IRN-diag, however, lead to an increase in RRE—an issue that could be remedied, should a stopping criterion for the outer iterations be imposed. The F-aTV method yields a poor reconstruction of the phantom, with lots of artifacts and incorrect scaling of the pixel intensities. Excluding FBTV and F-aTV, all the methods realize, on their final iteration, a reconstruction that has total variation similar to that of the true phantom’s (as can be seen in the bottom row of Figure 10).

**Figure 11:**
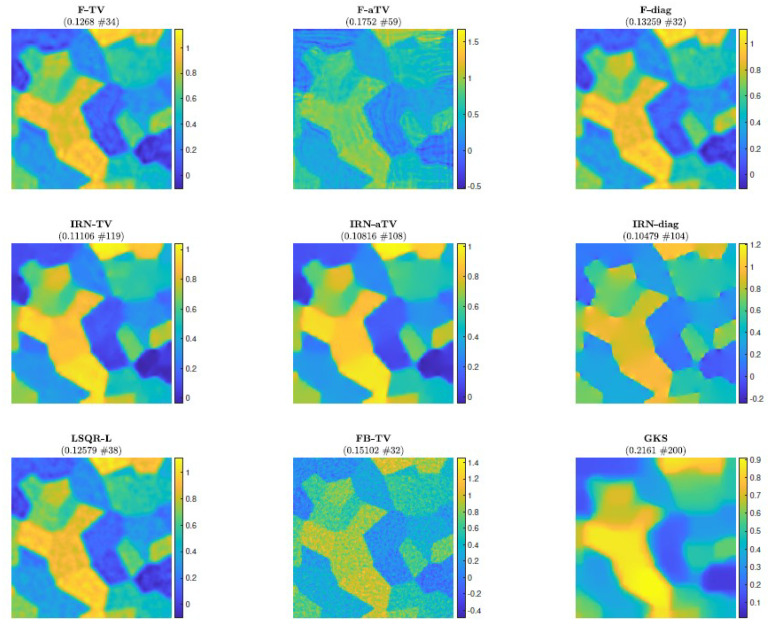
Experiment 3. Reconstructions achieved by various methods. The RRE and corresponding stopping iteration are displayed in the frame titles.

The reconstructions achieved when the stopping criterion is satisfied are displayed in Figure 11.

All the experiments considered in this section show that the new FGK-based solvers are indeed competitive with other popular edge-enhancing solvers: in particular, they achieve results of similar or improved quality with an increased speedup when it comes to the number of performed iterations. In all the examples, the reconstructions recover the edges and piecewise constant features of the exact images; the iteration-dependent weights are also able to recover rapidly changing and smooth features whenever they are present. The quality of the reconstructed solutions depends on the considered regularization terms and weightings, too: the performance of TV, aTV, and the edge-enhancing weights is obviously not the same across the considered test problems, but there is at least one such regularizer that, when considered within the FGK-based solver, delivers excellent results. For all these methods, the regularization parameters and stopping iteration are adaptively selected, leading to reliable parameter-free solvers.

## 6. Conclusions and Future Work

This paper introduced new solvers, based on a hybrid FGK iterative scheme, that can be efficiently employed to regularize and recover edges in inverse problems arising in imaging applications. The new solvers leverage an MM optimization approach and can handle different regularization terms expressed as iteratively reweighted 2-norms—namely, TV, anisotropic TV, and some heuristic edge-enhancing weights. The new solvers share the same theoretical framework as IRLS or IRN methods, and experimentally produce reconstructions of similar or better quality; however, they are inherently more efficient than IRN, since the inner–outer iterative schemes employed by IRN for solving the quadratic problems and updating weights are replaced by flexible Krylov methods that allow weights to update at each iteration, i.e., while the quadratic problems are solved. The regularization parameter can be set adaptively at each iteration, with negligible computational cost. The results of extensive numerical tests show that the new FGK-based solvers deliver solutions of similar or better quality even when compared with other state-of-the-art solvers for TV regularization, such as the forward–backward method.

Future work will focus on performing further theoretical analysis to provide alternative justifications for the use of the approximate pseudoinverse ${\tilde{L}}^{†}$, as well as building a solid theoretical framework for adaptive regularization parameter choice within flexible Krylov methods. Possible extensions will include handling high-order or fractional-order TV regularization terms [41,42], even in a 3D or tensorial framework [43], as well as regularized functionals that involve more than one regularization term. Such future investigations, if successful, may provide an efficient alternative to current regularization methods incorporating infimal convolutions of total-variation-type functionals [44], which are especially relevant when spatial and temporal regularization should be employed, e.g., in video processing.

## Figures and Tables

**Table 1:** Summary of the acronyms denoting various solvers considered in Section 5, and coherent marker and color codes used in most of the figures.

Solver	Acronym	Marker
‘priorconditioned’ LSQR with $L=D_{2d}$	LSQR-L	dashed blue
fast gradient-based TV	FBTV	magenta
generalized Krylov subspace method with isotropic TV	GKS	purple
FLSQR with isotropic TV utilizing approximation ${\tilde{L}}^{†}$	F-TV	red
FLSQR with isotropic TV utilizing ${\left(W_{K}D_{2d}\right)}^{†}$	F-TV(p)	–
FLSQR with anisotropic TV	F-aTV	circled purple
FLSQR with edge-enhancing weights	F-diag	light blue
IRN LSQR with isotropic TV	IRN-TV	yellow
IRN LSQR with anisotropic TV	IRN-aTV	green
IRN LSQR with edge-enhancing weights	IRN-diag	maroon

## Data Availability

The data as well as the software used to generate the numerical tests displayed in Section 5 of the present paper are publicly available through github, https://github.com/silviagazzola/EdgeEnhancingFGK, accessed on 25 September 2021.
